# Simulating the impact of methadone prescribing and pharmacy dispensing on opioid treatment and overdose in New York State: A study protocol for an agent-based modeling study

**DOI:** 10.1371/journal.pone.0335123

**Published:** 2025-10-22

**Authors:** Noa Krawczyk, Megan Miller, Ignacio Bórquez, Caroline Rutherford, Georgiy Bobashev, Pamela Mund, Katherine Keyes, Magdalena Cerdá, Ashly E. Jordan

**Affiliations:** 1 Center for Opioid Epidemiology and Policy, Department of Population Health, NYU Grossman School of Medicine, New York, New York, United States of America; 2 Department of Epidemiology, Mailman School of Public Health, Columbia University, New York, New York, United States of America; 3 Center for Data Science and AI, RTI International, Research Triangle Park, Morrisville, North Carolina, United States of America; 4 New York State Office of Addiction Services and Supports, New York, New York, United States of America; PLOS: Public Library of Science, UNITED STATES OF AMERICA

## Abstract

Amid the ongoing overdose crisis, U.S. lawmakers are considering policy reforms that could significantly change availability and accessibility of methadone treatment (MT) for opioid use disorder (OUD). However, uncertainty remains about which potential changes will lead to the greatest health benefits while minimizing unintended harms. In this protocol, we describe a planned NIH-funded study *(R21DA061660)* to simulate alternative MT delivery scenarios currently being considered in U.S. policy discussions, and estimate their impact on population-level rates of treatment initiation and retention and opioid overdose across different sociodemographic groups. We will use an agent-based model focused on 16 counties in NY State to simulate two alternative policy scenarios compared to the current status quo of opioid-treatment program (OTP) delivered MT: 1) office-based prescribing by addiction-certified providers with pharmacy and OTP dispensing; and 2) office-based prescribing by general practitioners with pharmacy and OTP dispensing. Agents will represent individuals with OUD and we will simulate access to MT based on alternative policy scenarios (e.g., locations of existing OTPs vs. provider offices and pharmacies). Probabilities of treatment initiation, retention, and opioid overdose will be informed by estimates from the scientific literature and administrative datasets from NY State. Multiple implementation scenarios will be considered to account for potential variation in adoption of office-based methadone by patients, providers, and pharmacies. To ensure relevance to directly impacted communities and policy makers, the study involves a collaboration between academic researchers and NY State government partners and relies on input from an Expert Advisory Board of people with lived and living experience with methadone, addiction medicine, and policy experts. Findings will be disseminated via a public dashboard. This study will inform ongoing policy discussions and shed light on the potential of researcher-policy partnerships to promote evidence-based policies that can reduce overdose and improve population health.

## Introduction

### Expanding methadone treatment for opioid use disorder is urgently needed to address the U.S. overdose crisis

The overdose epidemic continues to be a public health crisis in the U.S. Despite welcomed reductions in overdose deaths over the past two years, overdoses largely fueled by illicit opioids continue to kill over 80,000 people per year [1,2]. People with opioid use disorder (OUD) remain at greatest risk for opioid overdose, making expanding access to effective treatments for OUD an urgent policy priority. Methadone is a long-acting opioid agonist medication for OUD approved by the U.S. Federal Drug Administration [3]. Hundreds of studies worldwide have demonstrated its effectiveness in reducing illicit opioid use, improving health [3,4], and cutting overdose risk by over 50% [5,6]. Still, methadone treatment (MT) is vastly underutilized in the U.S: In 2020, only 311,531 of the estimated 7.6 million with OUD received MT [7,8]. As the U.S. illicit opioid supply has shifted almost entirely to fentanyl [2], the need to expand MT is greater than ever, as people who use fentanyl or have more severe OUD often report that methadone is more effective compared to other medications like buprenorphine [9,10].

Despite calls to expand use of MT to reduce overdose risk, the U.S. methadone regulatory framework and treatment delivery system continues to limit availability and access to MT. Between 2010 and 2019, MT utilization rose a mere 39% in the U.S., compared to a 222% rise in buprenorphine [7]. Unlike buprenorphine, which can be prescribed in office-based settings and dispensed in pharmacies, methadone – when used for OUD – can only be accessed via Opioid Treatment Programs (OTPs). Extensive regulations from the U.S. Drug Enforcement Administration (DEA) and Substance Abuse and Mental Health Services Administration (SAMHSA) have made it difficult to establish new OTPs [11,12]. State and local laws often impose additional restrictions on where OTPs can be located [13]. Due in part to these regulatory barriers, 80% of U.S. counties do not have OTPs, making MT highly difficult to access for many who need it [14]. Furthermore, there are geographic disparities in access to OTPs, with those residing in rural areas facing longer travel times as OTPs are more likely to be located in urban areas [15,16].

Beyond the limited availability of OTPs, the nature of the U.S. MT system acts as a deterrent to treatment initiation and retention. Stringent regulations that established and continue to govern OTPs are highly punitive and silo MT from other health services. Patients are mandated to visit OTP clinics often on a near-daily basis, with multiple prerequisites for service, such as drug testing and mandated counseling, and are often underdosed [17,18]. Decades of research document patient experiences in OTPs as stigmatizing and burdensome [19–21]. As OTPs were historically concentrated in racially minoritized and lower-income communities that lack access to alternative MOUD options (e.g., buprenorphine) [22], patients from marginalized groups often bear the greatest burden of OTP regulations, exacerbating racial disparities and stigma [23].

### Alternative U.S. policies could expand MT to office-based settings with pharmacy-dispensing, but evidence on potential outcomes is needed to inform policy decisions

For the first time in decades, U.S. lawmakers have begun considering policies that would expand MT delivery beyond OTPs to allow for office-based prescribing and pharmacy-dispensing. The ongoing overdose crisis, along with changes in MT delivery instigated by the COVID-19 pandemic [24] have led to calls to significantly reform the U.S. MT delivery system. Other countries, such as Canada, Australia, and the U.K., for example, use a combination of physician prescribing, pharmacy dispensing, and specialty clinics, approaches which have been shown to be safe and effective [11,25,26]. In 2024, a bipartisan bill supported by the American Society for Addiction Medicine – the “Modernizing Opioid Treatment Access Act (MOTAA),” was introduced in Congress that would allow physicians to prescribe and pharmacies to dispense MT outside of OTPs [27].

Research is urgently needed to understand the potential impact of newly proposed MT policies and how variation in adoption of such alternative MT models could impact OUD and overdose outcomes. Office-based methadone prescribing has never been introduced at scale in the U.S., but trials are ongoing [28] and past pilots and small programs have found these models to be successful and welcomed by patients [29–33]. Additionally, geospatial analyses show that expanding MT to physician offices and pharmacies would significantly alleviate travel burden among patients [16,34,35]. Still, there is much debate and uncertainty about the population-health impact of such policy changes, and whether the benefits (e.g., reduced overdose) outweigh potential risks (e.g., methadone diversion and inadvertent overdose) [36]. There are also questions about whether only board-certified addiction specialists or all licensed physicians (including general practitioners such as primary care providers) should be allowed to prescribe MT, which has important implications for reach and implementation of office-based MT [35,37]. Thus, there is a need for innovative research to quantify the impact of differential models of MT delivery on OUD treatment and overdose across multiple sociodemographic subgroups.

### Advanced computer modeling studies can help simulate the impact of alternative methadone policies on health outcomes

Ideally, randomized control trials of different MT policies would determine which lead to the greatest public health improvements. While such trials are not feasible, the field of complex systems science has developed computational tools that can help researchers emulate real-world complexity. One such tool, agent-based modeling (ABM), creates computer representations of systems comprising heterogeneous agents (i.e., individuals), their social networks, and the environment (e.g., health services) [38]. In ABM, “agents,” or simulated individuals from the target population, are endowed with a set of characteristics and parameters – based on real-world data and probabilities – that influence their behavior and interactions with each other and elements of the environment. They can “learn” from interactions and adapt their behavior over time. The stochastic nature of ABMs introduces elements of randomness, such that variability in potential population-level trends can be observed over multiple simulation runs to examine the range of potential outcomes given a set of specified conditions [39]. In addition, ABMs can incorporate other elements that may influence treatment outcomes (e.g., varying adoption of MT by patients/providers) and can inform decisions about best geographic locations for services (e.g., locations of MT access points) based on need. As ABMs incorporate demographic and geographic characteristics of the population, they allow for assessment of health outcomes across multiple population groups [39,40]. Results of these models – which can be visualized through animation – are useful for informing policy makers on the most promising strategies to maximize health outcomes.

ABMs are most relevant when they are tailored to a specific target population. Our team previously developed an ABM to help guide strategies to reduce overdose in New York State (NYS) as part of the NIH initiative “Healing Communities Study” (HEAL) [41]. Results of that model – the Simulation of Community-Level Overdose Prevention Strategies (SiCLOPS) – demonstrated the need for tailored county-level interventions focused on increasing naloxone and buprenorphine access, but did not explicitly focus on or examine MT strategies [41]. The current protocol describes the design and development of a novel iteration of this ABM – Simulating the Impact of Methadone Prescribing and Pharmacy Dispensing on Opioid Treatment and OverDose (SIMPOD) – to compare potential outcomes of alternative MT delivery scenarios on OUD treatment and fatal and non-fatal opioid overdose outcomes in NYS.

## Materials and methods

### Ethics statement

The NYU Langone Institutional Review Board reviewed this study and deemed it to be exempt from Human Subjects Research under Federal regulations Exemption 4.

### Setting

The current study aims to locally simulate the potential impact of alternative MT policies in NYS. NYS has an overdose rate similar to the national average [2], and the state is ethnically, racially, geographically and socioeconomically diverse. Nearly a quarter of the NYS population lives in rural counties [42], which have poor access to OTPs. [43,44] This geographic and sociodemographic variability makes it possible to simulate the impact of multiple MT delivery scenarios on health outcomes across multiple population groups. The ABM focuses specifically on 16 NYS counties originally calibrated for the NIH HEALing Communities initiative modeling study, of which NYS was one of four participating states. These counties were chosen because they represent diverse overdose and treatment landscapes, with an equal number of urban and rural counties, and thus able to represent an array of intervention dynamics [45]. The model purposefully excludes New York City, which is an MT anomaly in the U.S. due to large concentrations of OTPs.

### State government collaborators and Expert Advisory Board

Given the purpose of this study is to inform real-world MT policy and practice decisions, the study was designed in direct collaboration with the NYS Office of Addiction Services and Supports (OASAS), the agency that regulates and oversees substance use disorder services, including MT, for all state licensed specialty treatment facilities. The study leverages a multiple-principal investigator (mPI) model that involves an academic lead (Krawczyk) and government lead (Jordan), who jointly make decisions around study aims and directions. This partnership allows for tailoring study questions and dissemination activities to the needs of policy makers and practitioners in NYS and beyond. Additionally, this study is being guided by an Expert Advisory Board of eight individuals to ensure the ABM reflects the most likely scenarios and pressing policy questions, and that findings reach diverse stakeholder groups. The Board purposefully comprises people with lived and living experience with MT and who hold leadership positions in organizations that advocate for the needs of people on methadone and people who use drugs, such as the National Survivors Union and the National Coalition to Liberate Methadone. Given the historical marginalization of people who use drugs and the particular exclusion of people on methadone from treatment and policy decisions, this was deemed critical to the purpose and value of this work. Additional Board members include individuals who serve in state government roles overseeing OUD treatment, hold leadership positions in addiction medicine and treatment committees, and who have expertise in OUD policy, economics, and/or simulation modeling. The Board will meet twice a year over the study period to advise on model structure, inputs, and adoption levers, help interpret study findings and implications, and inform dissemination strategies to ensure the study reaches wide audiences, including directly impacted communities.

### Overview of simulated policy scenarios and study objectives

**Fig 1:**
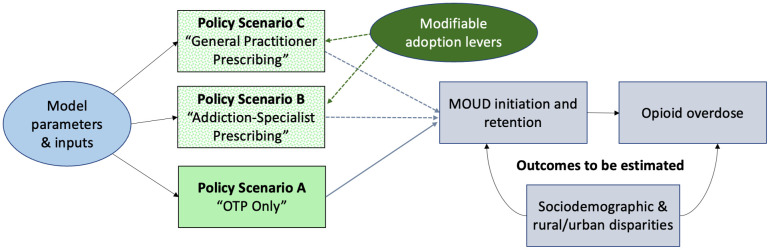
Overview of policy scenarios and study outcomes. Note: Dotted lines and boxes represent hypothetical scenarios that are not currently available.

The overall goal of this study is to compare potential outcomes of alternative MT delivery policy scenarios on OUD treatment and overdose outcomes. As described below and shown in Fig 1, we will focus on three specific policy scenarios:

- **Scenario A: OTP Only (reference).** Assumes no changes in the current MT landscape. Here, OTPs are the sole providers/dispensers of MT.
- **Scenario B: Addiction-Specialist Prescribing.** Assumes MT landscape proposed by 2024 MOTAA bill, in which OTPs and addiction-board providers act as primary prescribers of MT, with dispensing allowable via OTPs and community pharmacies. We will use registries of licensed addiction medicine and addiction-psychiatry board certified providers [46] to emulate which providers will be available to adopt MT prescribing.
- **Scenario C: General Practitioner Prescribing.** Assumes MT landscape in which OTPs and any medical provider with an active DEA registration can prescribe MT, with dispensing via pharmacies and OTPs. This scenario reflects a more drastic expansion of MT endorsed by some advocacy groups [25,26]. We will use data on buprenorphine-waivered providers [47] and non-OTP specialty treatment programs that currently prescribe buprenorphine [48] to emulate the general practitioners most likely to adopt MT prescribing.

For each scenario, we will model how differential policies and subsequent access points to methadone prescribing and dispensing impact MOUD initiation and one-year retention, opioid fatal and non-fatal overdose, and how these outcomes are experienced differently among those from different sociodemographic groups and rural vs. urban counties. Table 1 includes information on what data sources will be used to inform MT prescribing and dispensing locations under different policy scenarios.

**Table 1:** Data sources used for model inputs, parameterization, and calibration.

Model Element	Data Source
**Agent Characteristics and Location**	
Demographics (age, sex, race/ethnicity, household income, geographic coordinates of household)	2019 RTI SynthPop Synthetic Population Dataset [49]
Probability of OUD by sex and age group	2019 Bayesian evidence synthesis prevalence estimates for NY Counties [50]
**Methadone Treatment Prescribing and Dispensing Locations**	
Licensed OTPs (Policy Scenario A, B, C)	2025 NYS Office of Addiction Services and Supports Specialty Treatment (OASAS) Data [48]
Addiction-board certified providers (Policy Scenario B, C)	2025 American Medical Association Data on Addiction Medicine and Addiction Psychiatry Board Certifications [46]
General practitioners waivered to prescribe buprenorphine (Policy Scenario C)	2024 SAMHSA Buprenorphine Provider Locator Tool [51]
OASAS licensed treatment programs that are not OTPs but offer buprenorphine prescribing (Policy Scenario C)	2025 NYS Office of Addiction Services and Supports Specialty Treatment (OASAS) Data [48]
Outpatient pharmacies (Policy Scenario B, C)	2025 Medicaid Enrolled Pharmacy Data from NYS Department of Health [52]
**Probability estimates for outcomes of interest**	
Methadone treatment initiation/retention	2019-2023 NYS Office of Addiction Services and Supports Specialty Treatment (OASAS) Data [48]
Buprenorphine and extended-release naltrexone initiation/retention	2019 IQVIA Pharmacy Prescriptions Data for NY counties [53]
Non-fatal opioid overdose hospitalizations	2019-2023 Healthcare Cost and Utilization Project (HCUP) data for hospitalizations in NY counties [54] and peer-reviewed literature.
Fatal opioid overdoses	2019-2023 Vital Statistics Data for overdose deaths in NY counties [55] and peer-reviewed literature.
Methadone diversion and associated overdose	Peer-reviewed literature [6,56–63,64–66]

### Model design

**Fig 2:**
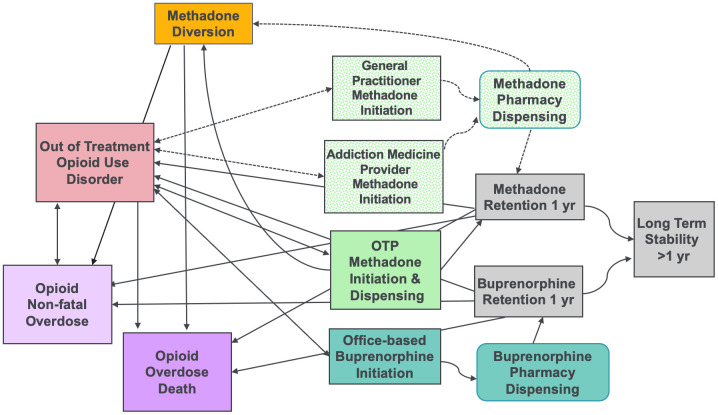
ABM Conceptual Model. Note: Dotted lines and boxes represent hypothetical scenarios that are not currently available.

The ABM structure and relationships are derived from a conceptual model for how agents (i.e., individuals with OUD) transition between being out-of-treatment to entering and/or staying in MOUD treatment, and how each of these states impacts fatal and non-fatal opioid overdose risk (Fig 2). ABMs enable the characterization of elements – and interactions between elements – of a complex and modifiable system rather than examining isolated features of a stagnant system [67]. This model structure was adapted from the prior NY SiCLOPS ABM [41] and incorporates novel information on MT access and dispensing, modeling both existing scenarios noted as solid lines (moving from out-of-treatment to OTP) and hypothetical scenarios based on alternative policies noted as dotted lines (moving from out of treatment to office-based MT with pharmacy dispensing). Given the importance of considering methadone risks when making policy decisions– particularly the potential for diversion or overdose with methadone [56] – we will also model the impact of potential diversion and subsequent overdose risk associated with different MT settings. Access to and use of buprenorphine and extended-release naltrexone will be incorporated into the model as an important part of the treatment landscape and modifier of overdose risk. We will model OUD treatment utilization and opioid overdose through common individual (e.g., demographics) and environmental factors (e.g., distance to MT providers and pharmacies). Each target outcome will be calibrated to multiple administrative data sources from NYS as described below. Although models like this could become complex, we will use standard Overview, Design Concepts and Detail (ODD) protocols that clearly describe the structure, design, and functioning of the model for transparency and replicability [68].

### Data sources

Multiple data sources will be used to model MT access points within each scenario, as well as to parameterize and calibrate the model. Data sources were selected to provide the most granular and specific estimates for the 16 NYS counties that are being modeled. Given the importance of considering individual residence and distance to treatment and how it impacts differential access to MT under different policy scenarios, we will use the open-source RTI SynthPop Synthetic Population Dataset, developed by co-authors on this project [49], which contains individual-level data representative of real people with spatially explicit records within every U.S. household. In parallel, we will leverage Bayesian evidence synthesis-derived OUD prevalence estimates for counties in NY State, developed by co-authors of this study, to estimate the distribution by sex and age group of OUD across our synthetic population of agents in each county [50]. Wherever possible, we will analyze administrative local data from NYS to inform model inputs and calibration targets (e.g., NYS data on opioid overdose hospitalizations, opioid overdose deaths, buprenorphine/naltrexone prescriptions). For example, to derive initiation and retention probabilities that best reflect the NYS population, we will use individual-level de-identified data on MT initiation and retention from administrative data collected and maintained by OASAS. When local NYS data are not available (e.g., estimates on methadone diversion), we will rely on the peer-reviewed literature and systematic reviews/meta-analyses. Table 1 summarizes our expected model input strategy with each respective data source. When reporting findings, we will share all model input values to ensure replicability and transparency.

### ABM parameterization, calibration, and validation

Once all data sources are gathered, each entity and relationship in the ABM must be assigned at model initialization a static or dynamic parameter that may be dependent on other model parameters. The physical environment will be represented using Geographic Information Systems (GIS) shapefiles that geo-locate agents within households, OTPs, office-based addiction treatment providers, and pharmacies. This will allow us to model modifiable distances to methadone prescribers and dispensers across the different policy scenarios. Agents will be simulated to reflect the population of individuals with OUD in each county by sex and age group. In addition, agent will have different MOUD initiation/retention and opioid overdose probabilities based on basic (e.g., age, sex, race/ethnicity) and dynamic characteristics and behaviors (e.g., deaths, births, diversion, distance), derived from conditional probability distributions that are updated over each time step in model runs based on simulated scenarios. Characteristics of individuals and their parameters will be derived from multiple data sources specific to NY counties, where possible, as well as findings from prior research when local data do not exist or are unavailable. Model estimates of each target will be calibrated to match trends in annual county-level data points. We will validate the model prior to manipulating experimental parameters to ensure that dynamic interactions of agents reproduce observable phenomena based on empiric data (e.g., overdoses, treatment entry) [67]. To do this, first, we will test that birth, death, and migration dynamics of the agent population mirror the vital statistics and residential stability of NYS. Second, we will compare predicted temporal trends in outcomes (MOUD initiation, retention, non-fatal, and fatal opioid overdoses) under current policy conditions (“OTP only”) with empirical estimates using NYS data from 2019 to 2023. We will calibrate accordingly to match the observed targets. Third, we will determine the impact of varying key parameters to test the model’s robustness to variations in model assumptions. Throughout the calibration and validation process, we will regularly engage in qualitative assessment amongst our team members and Expert Advisory Board to ensure the model meaningfully represents real-world dynamics. The ABM will be written and calibrated in NetLogo v.6.4.0. [69] and analyses to inform model inputs will be conducted using R (version 4.4.3) [70].

### Simulating health outcomes for each policy scenario

Once the ABM is calibrated to mimic observed phenomena, we will modify elements of the environment of the ABM to simulate those of the proposed MT policy scenarios, reflecting the varying MT geographic access points. We will simulate the following health outcomes over a five-year period (2019–2024) across the different policy scenarios:

- MT initiation: *To simulate changes in MT initiation*, we will model initiation in MT among agents based on sociodemographics and proximity to prescribing access points (e.g., OTPs/office-based providers). We will use OASAS data combined with synthpop simulated data on OUD prevalence to estimate initiation rates based on driving distance to OTPs. We will additionally incorporate estimates from the scientific literature on the relationship between MT driving times and treatment utilization to simulate how initiation may change as distance to prescribing points changes.
- MT retention: *To simulate changes in MT retention*, we will model retention in MT among agents based on sociodemographics and proximity to dispensing access points (e.g., pharmacy dispensing). We will use current OASAS data on retention rates based on driving distance to OTPs to simulate how retention changes as distance to dispensing points changes. We will further complement data from the State with estimates from published literature (e.g., relative retention among those in OTPs vs. office-based MT in clinical trials) [71].
- Methadone diversion: To simulate changes in methadone diversion, we will incorporate estimates of diversion likelihood and subsequent overdose risk by treatment location type based on studies from the existing literature that were synthesized in a recent narrative review conducted by our team [56].
- Fatal overdose and non-fatal overdose hospitalizations: We will use the findings on initiation, retention, and diversion to estimate changes in non-fatal opioid overdose hospitalization rates and opioid overdose death rates. These rates will consider changes to MT initiation and retention, incorporating the known overdose risk reduction estimates of MT use [6] assuming constant availability of other existing interventions for opioid overdose (e.g., buprenorphine, extended-release naltrexone, and naloxone). These estimates will also incorporate any increased overdose risk derived from changes to estimated diversion based on MT access changes [72].
- Estimating differences across distinct sociodemographic groups: For each outcome, we will stratify analyses by agent sociodemographic characteristics (e.g., age, sex race/ethnicity) and rural vs. urban geography of the county (based on NYS rurality definitions) [42] to estimate differential effects of each scenario on different population groups, and assess which scenarios result in increased vs. decreased health disparities.

### Accounting for policy adoption and impact uncertainty

Within each of the hypothetical policy scenarios B and C that have not been implemented in the U.S. and for which we do not have administrative data, it is important to acknowledge a wide degree of uncertainty about rates of MT adoption by office-based providers and pharmacies. For instance, it is likely that even if allowable by law, not all providers will become MT prescribers, and not all pharmacies will become MT dispensers. There is also uncertainty about what proportion of patients will be eligible for office-based prescribing and pharmacy dispensing under new policy scenarios. To model this uncertainty, we will build in multiple “*levers*” that allow us to estimate outcomes under various circumstances of adoption and uptake. This will help identify implementation scenarios with the greatest reductions in overdose. For example, we will first develop “ideal” scenarios, in which all eligible providers and pharmacies participate in MT prescribing and dispensing, and then model the extent to which any benefits from additional access points are reduced based on lower adoption scenarios (e.g., only 25% of pharmacies dispense MT). Similarly, we will model differential outcomes based on alternative policy scenarios in which all vs. only some patients (e.g., those in treatment for 3 + months) become eligible for office-based methadone prescribing. Additionally, given office-based MT for OUD is not available in the U.S., we do not have local estimates on how office-based MT may differentially impact initiation and retention relative to OTPs. We will thus begin by assuming similar initiation and retention in MT based on travel time to access points, and then model if and how outcomes would change if patients’ initiation and retention rates improved or worsened based on treatment setting. For all modeling decisions around extent of adoption or impact, we will rely on published peer-reviewed literature from the U.S. and elsewhere, as well as feedback from government partners and Expert Advisory Board to ensure modeling assumptions and ranges reflect realistic and well-supported scenarios.

### Status and timeline

As of submission of this manuscript in September of 2025, we have begun gathering data sources and scientific literature that will inform our parameters and calibration targets. We expect data collection to be complete by the end of 2025, with calibration and validation conducted in early 2026. Primary results of this model are expected in June/July of 2026, with dissemination activities to take place throughout the rest of 2026.

## Discussion

Our study presents an example of how computer simulation models can be designed to directly inform policy decisions for which real-world outcomes data are not yet available. Indeed, ABMs have been successfully used to guide policy and programs for issues as diverse as infectious diseases, violence, and overdose [39,73], but none to our knowledge have simulated the impact of alternative MT policies on health outcomes. The current study will apply an ABM approach to simulate various MT policy scenarios using data specific to a local setting and population. Additionally, our model will build in levers to simulate various levels of adoption and uptake of alternative MT models of care, which can guide implementation efforts towards achieving the best outcomes if or when new policies go into effect.

The key to the success of this project in informing policy conversations around MT in the U.S. will be our ability to effectively disseminate findings to a wide range of audiences, including directly impacted patient groups, state and federal policy makers, addiction treatment providers and programs, and the broader public, for which MT is still a highly controversial topic [74]. In addition to disseminating findings via peer-reviewed publications and conferences, we will create a public-facing dashboard linked to the NYS OASAS website that will allow interested individuals and policy makers to observe simulated outcomes under variable policy and adoption scenarios. Once the dashboard is created, we will also engage in multiple efforts to disseminate findings via drug policy listservs, drug user advocacy groups, addiction care and MT provider associations via webinars and targeted outreach. Finally, we will continue to build on this work in future research by expanding and replicating the model to other states and diverse jurisdictions with unique overdose and OUD treatment characteristics, and update our models as the overdose crisis and priorities evolve.

This study is subject to multiple limitations. First, our model will be limited to simulating the impact of alternative MT policies in 16 counties of NY State based on local data, and findings may not be readily generalizable to other settings. However, the relationships modeled (e.g., risk of opioid overdose based on treatment setting, influence of geographic access to MT and treatment initiation, diversion likelihood) are not unique to NYS, and findings may therefore still be informative for other jurisdictions and national conversations on the topic. Second, while we will utilize rich local data on our outcomes of interest, including treatment utilization and overdose data, and rely on an extensive peer-reviewed literature to inform our model parameters, some data and estimates may not be available or necessarily transportable to our local context and scenarios. Where possible, we will conduct sensitivity analyses to account for uncertainty of our estimates and assumptions. Third, while our model will be informed by the most recent data and calibration targets available, the overdose crisis is rapidly evolving, and risks of overdose and treatment success may depend on changes in the illicit market and cohort effects and norms. We will update our model to calibrate targets using new data to the extent they become available throughout the study period, and include additional model components to improve performance. Finally, the MT landscape is extremely complex, and treatment utilization, outcomes, and risks are multifactorial and depend on a variety of individual, practice, and structural-level factors. Given our goal of efficiently generating and disseminating findings to inform timely policy decisions, we will not be able to model all of this complexity and must make simplifying assumptions for the sake of building a parsimonious model in a short timeframe. Still, all decisions will be carefully informed by our expert team and Expert Advisory Board to ensure modeling inputs are backed by evidence and calibrated and validated to ensure robustness and reflect real-world scenarios and outcomes.

## Conclusion

MT reform is long overdue in the U.S., but the right way forward remains a fierce topic of debate amongst policy makers, treatment providers, and patient advocacy groups [75] . The current simulation project has potential to generate quantitative data that can inform these decisions beyond ideology and opinion. Critical to the success of this effort is our unique academic-government partnership and our purposeful integration of lived experience, policy, and addiction medicine feedback. Without such collaborations and intentional dissemination plans, simulation studies risk being a mere intellectual and academic exercise with questionable influences on public health policy or practice. As the nation continues to grapple with tens of thousands of opioid-related overdoses every year, it is the hope that findings from our ABM can inform national and local discussions to promote evidence-based policies that achieve the greatest reductions in overdose and improvements in health.

## Abbreviations

OUD: Opioid use disorder
MOUD: Medications for opioid use disorder
NYS OASAS: New York State Office of Addiction Services and Supports
MT: Methadone treatment
OTP: Opioid treatment program
MOTAA: Modernizing Opioid Treatment Access Act.
